# Trends and cross-country inequalities in the global burden of subarachnoid hemorrhage, 1990–2021: a population-based study

**DOI:** 10.3389/fpubh.2026.1672856

**Published:** 2026-05-04

**Authors:** Shuai Li, Li Li, Shengnan Zhang, Xin Chen, Tianyu Chen, Hongpeng Liu

**Affiliations:** 1 Xin’an Traditional Chinese Medicine Clinic, Anhui Province, Hefei City, China; 2 Yunnan University of Traditional Chinese Medicine, Kunming, Yunnan, China; 3 The Third Affiliated Hospital of Yunnan University of Traditional Chinese Medicine, Kunming, Yunnan, China

**Keywords:** subarachnoid hemorrhage, global burden of disease, SDI, disease burden, predictive analysis

## Abstract

**Objective:**

This study aimed to analyze global and regional trends in the burden of subarachnoid hemorrhage (SAH) from 1990 to 2021, focusing on incidence, prevalence, disability-adjusted life years (DALYs), and mortality, and to examine socioeconomic inequalities across 204 countries and territories using Global Burden of Disease (GBD) data.

**Methods:**

Utilizing GBD 2021 data, we assessed SAH burden by incidence, prevalence, DALYs, and mortality, stratified by Socio-Demographic Index (SDI), sex, and region. Age-standardized rates were calculated to ensure comparability. Socioeconomic inequalities were evaluated using the Slope Index of Inequality (SII) and Concentration Index (CII). Spearman correlation analyses explored associations between SAH burden and SDI.

**Results:**

From 1990 to 2021, global SAH incident cases increased from 508,789 (95% UI 441,504–587,616) to 697,486 (95% UI 614,334–795,785), and prevalent cases rose from 4.90 million (95% UI 4.42–5.39 million) to 7.85 million (95% UI 7.16–8.58 million). Age-standardized incidence, prevalence, DALY, and mortality rates declined significantly, from 11.69 to 8.33, 109.90 to 92.17, 275.85 to 125.20, and 9.54 to 4.18 per 100,000, respectively. East Asia showed the largest declines (e.g., incidence from 17.74 to 7.89 per 100,000), while Andean Latin America had the highest burden (2021 prevalence: 172.03 per 100,000). Females had higher incidence and prevalence but lower DALYs and mortality. Middle SDI regions exhibited the largest case increases and rate declines. Socioeconomic inequalities worsened, with 2021 DALY SII at −47.27 (95% CI −71.58, −22.97) and mortality SII at −1.33 (95% CI −2.14, −0.51), indicating a shift in burden toward lower-income groups. SAH burden showed weak negative correlations with SDI, stronger for DALYs (*r* = −0.3541, *p* < 0.001) and mortality (*r* = −0.3058, *p* < 0.001).

**Conclusion:**

Despite increasing absolute SAH cases due to population growth and aging, age-standardized rates declined, reflecting improved risk factor management and healthcare. However, persistent regional disparities and growing socioeconomic inequalities, with heavier burdens in low SDI regions and lower-income groups, underscore the need for targeted interventions, enhanced healthcare access, and further research into region-specific risk factors to reduce the global SAH burden and address health inequities.

## 1 Introduction

Subarachnoid hemorrhage (SAH) is a severe medical condition characterized by bleeding into the subarachnoid space, typically caused by ruptured cerebral aneurysms or trauma, exerting a significant impact on global health (1). Despite advancements in medical care, SAH remains a major cause of disability and mortality worldwide, contributing substantially to the burden measured by disability-adjusted life years (DALYs) and premature mortality. The global epidemiology of SAH varies across regions, socioeconomic levels, and populations, influenced by factors such as healthcare access, prevalence of risk factors, and population aging (2). Previous Global Burden of Disease (GBD) studies have highlighted regional differences in SAH incidence, prevalence, and outcomes, yet comprehensive analyses of long-term trends and socioeconomic inequalities remain limited. Previous reports were limited to analyses spanning 1990–2019 (3), and the two most recent publications exhibit significant limitations, notably, the study Global, Regional, and National Burden of Subarachnoid Hemorrhage: Trends From 1990 to 2021 and 20-Year Forecasts (4) lacks comprehensive global regional analysis and provides insufficient examination of SDI regions, failing to adequately explore the non-correlation between SAH burden and SDI stratification observed in our findings. Similarly, Epidemiological trends of subarachnoid hemorrhage at global, regional, and national level: a trend analysis study from 1990 to 2021 (5) reports only incidence and prevalence rates without presenting absolute case counts (incident or prevalent cases) and omits analysis of inequalities, thereby remaining confined to descriptive statistics without detailed comparative studies or correlation analyses. In contrast, leveraging GBD data from 1990 to 2021, the present study analyzes global and regional trends in SAH burden—encompassing incident cases, prevalent cases, DALYs, and deaths, alongside their corresponding rates (incidence, prevalence, DALY rate, and mortality)—while further extending the analysis to investigate socioeconomic inequalities across 204 countries and territories (6). By assessing differences in Socio-Demographic Index (SDI), sex, and geographical regions, this study aims to elucidate SAH’s epidemiological characteristics, identify high-risk populations, and provide evidence for targeted public health interventions to mitigate its global impact.

## 2 Materials and methods

### 2.1 Data acquisition

SAH burden data were extracted from the GBD Public Database^1^ and exported in CSV format. Under the “cause” section, the “Select all GBD regions” module was chosen, encompassing all countries and territories globally. This included data from the following SDI regions: Global, Low SDI, Low-middle SDI, Middle SDI, High-middle SDI, and High SDI.

### 2.2 Key concepts

Regional Classification: The GBD study categorizes the world into 21 geographical regions to enable precise analysis of health trends and disease burdens across different areas.

SDI Framework: “The SDI classifies regions based on composite health, education, and economic metrics (GBD Study). Countries were categorized into five tiers: Low SDI (0–0.4658), Low-middle SDI (0.4658–0.6188), Middle SDI (0.6188–0.7120), High-middle SDI (0.7120–0.8103), and High SDI (0.8103–1.0000).

Age Stratification: Age groups in GBD are stratified into various intervals, with 5-year age groups being the most frequently utilized.

Age Standardization: Rates were age-standardized to eliminate the confounding effects of differing population age structures when comparing prevalence, incidence, mortality, and other metrics across populations.

Inequality Slope Index (SII): An absolute indicator for measuring the degree of health inequality, reflecting the linear relationship between the distribution of health indicators in socioeconomic status and socioeconomic status. When calculating, different regions are sorted from low to high according to their socioeconomic status, and relative ranking values are assigned to each group. Linear regression analysis is conducted on health indicators, and the slope of the regression model is SII.

Concentration index (CI): A relative indicator for measuring the degree of health inequality, reflecting the distribution of health indicators in terms of socioeconomic status. The value range is from −1 to 1. The closer the value is to 0, the smaller the degree of inequality. A positive value indicates better health among the affluent group, while a negative value is the opposite.

### 2.3 Data analysis

#### 2.3.1 Subarachnoid hemorrhage burden analysis

SAH burden data were imported into the JD_GBDR_V2.36 software (Anhui Data-Bio Information Technology Co., Ltd., China). The “Table Results” function was employed to output the prevalence, incidence, and DALY rates of SAH in China, globally, and across SDI regions for the years 1990 and 2021, facilitating a comparative analysis of the 2021 burden.

This study compiled data on SAH prevalence, incidence, SAH-related mortality, and SAH-related disability-adjusted life years (DALYs), encompassing their rates globally, regionally, and nationally.

Key variables derived from these data included year, age, sex, and location. Specific metrics were calculated as follows: Incidence Rate (per 100,000): Number of new cases divided by the population size. Prevalence Rate (per 100,000): Number of existing cases (new + prevalent) divided by the observed population during the same period. Mortality Rate (per 100,000): Annual number of deaths divided by the total population. DALYs: Quantified the impact of SAH, representing the number of healthy life years lost per 100,000 population annually. These metrics enabled consistent assessment of disease burden across different regions.

#### 2.3.2 Projection of SAH burden (2022–2050)

SAH burden data for China and globally were imported into R software (version specified if applicable) for processing. Utilizing the table package, a Bayesian age-period-cohort (BAPC) model was applied to analyze the long-term effects of age, period, and cohort on the disease burden. This model was used to project SAH burden trends for the global population from 2022 to 2050.

BAPC Models for Projections: (Bayesian Age-Period-Cohort) models provide a comprehensive framework for making projections using integrated nested Laplace approximations (INLA) for full Bayesian inference. Key features of BAPC models include: 1. Generation of age-specific and age-standardized projected rates. 2. Automatic addition of Poisson noise when interest lies in the predictive distribution. BAPC is particularly useful for projecting future rates based on historical data, making it an invaluable tool for public health planning and analysis (Analysis Tools Help Page).^2^ For detailed parameters and explanations, refer to the article (28139001) (7).

## 3 Results

### 3.1 Global and regional disease burden

This study utilized data from the GBD database, including global data, data from five SDI regions, and 21 key regions, to comprehensively compare incidence, prevalence, DALY rates, and mortality across 27 regions (Table 1). Analysis of GBD data revealed a significant increase in the global incidence of SAH from 1990 to 2021. The total number of incident cases rose from 508,789 (95% UI 441,504–587,616) in 1990 to 697,486 (95% UI 614,334–795,785) in 2021. By sex, male cases increased from 243,711 (95% UI 209,696–281,370) to 340,845 (95% UI 298,157–388,882), and female cases from 265,078 (95% UI 229,342–305,773) to 356,641 (95% UI 315,063–408,979). Among SDI regions, middle SDI regions showed the most significant increase, from 175,733 (95% UI 150,601–204,265) to 239,549 (95% UI 209,800–276,045). High SDI regions saw an increase from 102,828 (95% UI 89,935–120,062) to 130,354 (95% UI 116,018–148,269). Notably, South Asia exhibited the largest increase, from 79,739 (95% UI 68,837–92,811) to 140,534 (95% UI 120,483–162,708).

**Table 1:** Global and regional disease burden of Subarachnoid hemorrhage from 1990 to 2021.

Burden of disease	Location	All-age cases (95% UI)		Age-standandised incidence rates (95% UI)		PC
		1990	2021	1990	2021	1990–2021
Incidence	Global	508789.24(441504.10,587616.26)	697486.49(614334.16,795785.26)	9.54(8.28,11.02)	8.84(7.78,10.08)	−7.35(−10.65,-3.82)
	High SDI	102828.30(89934.94,120061.76)	130353.58(116017.76,148268.88)	11.69(10.23,13.65)	11.91(10.60,13.55)	1.91(−1.85,6.12)
	High-middle SDI	116209.33(101162.87,133959.43)	127288.04(113021.06,145761.03)	10.93(9.51,12.60)	9.76(8.67,11.18)	−10.67(−14.13,-6.85)
	Middle SDI	175732.54(150601.13,204265.03)	239549.13(209799.96,276045.38)	10.20(8.74,11.86)	9.78(8.57,11.27)	−4.08(−10.11,0.94)
	Low-middle SDI	85513.92(74422.07,98218.16)	145849.92(127059.46,166815.27)	7.36(6.41,8.46)	7.59(6.61,8.68)	3.11(−0.14,6.73)
	Low SDI	28028.78(24253.75,32454.39)	53832.90(46819.43,61525.96)	5.59(4.84,6.47)	4.82(4.19,5.51)	−13.83(−16.38,-11.03)
	Andean Latin America	4545.34(4013.73,5073.05)	7842.22(7001.16,8775.50)	11.96(10.56,13.35)	11.86(10.59,13.27)	−0.88(−5.32,3.98)
	Australasia	1690.91(1514.22,1900.73)	2630.46(2331.76,2987.10)	8.34(7.47,9.37)	8.50(7.53,9.65)	1.88(−4.88,9.80)
	Caribbean	3431.45(3012.36,3842.23)	5327.43(4776.88,6036.08)	9.72(8.54,10.89)	11.23(10.07,12.72)	15.45(9.61,21.58)
	Central Asia	4949.84(4311.61,5538.68)	7813.69(6959.81,8734.34)	7.14(6.22,7.99)	8.16(7.26,9.12)	14.20(9.90,18.61)
	Central Europe	12991.17(11527.10,14599.94)	12712.05(11517.73,13976.87)	10.39(9.21,11.67)	11.03(9.99,12.13)	6.19(1.81,10.76)
	Central Latin America	13686.95(12039.77,15342.66)	28466.30(25351.02,32267.74)	8.32(7.32,9.33)	11.25(10.02,12.75)	35.15(28.02,42.71)
	Central Sub-Saharan Africa	2483.41(2115.18,2915.41)	6071.30(5215.34,7066.56)	4.52(3.85,5.30)	4.43(3.81,5.16)	−1.87(−5.70,2.60)
	East Asia	154857.47(132434.16,181540.89)	151816.04(131564.62,176502.42)	12.72(10.88,14.91)	10.31(8.93,11.98)	−18.96(−25.41,-12.96)
	Eastern Europe	25111.35(21514.77,29212.88)	27737.13(24527.77,31834.86)	11.09(9.50,12.90)	13.42(11.86,15.40)	21.00(15.29,27.39)
	Eastern Sub-Saharan Africa	9670.05(8318.49,11211.48)	18583.86(15961.96,21445.30)	5.07(4.36,5.88)	4.36(3.75,5.03)	−13.93(−16.07,-11.56)
	High-income Asia Pacific	34840.30(30002.67,41206.30)	46617.76(41127.96,53645.35)	20.09(17.30,23.77)	25.14(22.18,28.93)	25.10(18.07,33.41)
	High-income North America	22730.38(19663.80,26711.35)	33649.14(29722.22,38721.69)	8.08(6.99,9.49)	9.09(8.03,10.46)	12.54(7.17,19.66)
	North Africa and Middle East	20475.20(17698.26,23128.07)	32386.33(28114.06,36307.39)	6.04(5.22,6.82)	5.20(4.51,5.83)	−13.88(−17.61,-9.31)
	Oceania	608.53(531.74,686.08)	1269.29(1123.11,1415.84)	9.29(8.12,10.47)	9.11(8.06,10.17)	−1.91(−5.80,2.63)
	South Asia	79739.43(68836.87,92811.38)	140533.75(120483.38,162708.02)	7.29(6.30,8.49)	7.61(6.52,8.81)	4.36(0.56,9.21)
	Southeast Asia	42836.32(37087.53,48904.56)	74812.40(65465.59,85579.57)	9.20(7.97,10.51)	10.71(9.37,12.26)	16.42(12.45,20.54)
	Southern Latin America	7571.59(6754.65,8607.98)	8417.52(7546.51,9475.44)	15.28(13.64,17.38)	12.43(11.15,14.00)	−18.64(−23.26,-14.13)
	Southern Sub-Saharan Africa	2096.41(1791.14,2422.57)	3590.21(3112.57,4139.79)	4.00(3.42,4.62)	4.47(3.88,5.16)	11.79(7.64,16.37)
	Tropical Latin America	16719.34(14083.88,19461.91)	26001.40(22460.43,30289.82)	10.96(9.23,12.76)	11.43(9.87,13.31)	4.27(−2.20,11.22)
	Western Europe	39375.98(34657.67,45305.84)	43945.23(39397.98,49547.92)	10.24(9.02,11.79)	10.05(9.01,11.33)	−1.91(−5.64,1.88)
	Western Sub-Saharan Africa	8377.83(7164.59,9575.58)	17262.99(14764.82,19802.45)	4.34(3.71,4.96)	3.52(3.01,4.04)	−18.74(−20.66,-16.72)
Prevalence	Global	4901711.27(4420922.37,5394921.87)	7852792.32(7164804.06,8578767.27)	91.90(82.89,101.15)	99.51(90.79,108.71)	8.28(6.02,10.43)
	High SDI	1228023.45(1117291.04,1344004.60)	1936625.81(1780962.47,2087719.88)	139.62(127.03,152.81)	177.01(162.79,190.83)	26.78(24.07,29.44)
	High-middle SDI	1029087.17(923783.90,1138888.35)	1313530.32(1193923.70,1448151.14)	96.76(86.86,107.09)	100.73(91.56,111.05)	4.10(1.59,6.51)
	Middle SDI	1541691.28(1374560.09,1702954.66)	2493467.12(2255823.48,2751504.25)	89.48(79.78,98.84)	101.83(92.13,112.37)	13.80(10.23,17.18)
	Low-middle SDI	805736.60(723602.42,890611.56)	1502241.92(1368010.49,1645542.09)	69.38(62.30,76.68)	78.20(71.21,85.66)	12.71(10.31,15.11)
	Low SDI	291964.65(264037.75,321771.06)	599906.76(550045.26,653032.23)	58.24(52.67,64.19)	53.69(49.23,58.44)	−7.82(−9.57,-5.99)
	Andean Latin America	53338.88(50555.06,56206.73)	108449.33(103325.03,113846.19)	140.39(133.06,147.94)	163.99(156.24,172.15)	16.81(13.95,19.45)
	Australasia	19830.45(18428.73,21454.01)	35127.02(32840.44,37589.91)	97.80(90.89,105.81)	113.45(106.07,121.41)	16.00(11.40,20.35)
	Caribbean	44260.03(41671.32,46948.68)	71255.78(67508.58,74882.82)	125.41(118.07,133.03)	150.14(142.25,157.79)	19.72(17.35,22.22)
	Central Asia	48426.80(44898.28,52110.68)	78142.32(73006.78,83491.46)	69.87(64.78,75.18)	81.56(76.20,87.14)	16.74(14.21,19.50)
	Central Europe	126143.22(115978.44,137367.86)	113644.85(104927.57,123406.14)	100.84(92.71,109.81)	98.60(91.03,107.06)	−2.23(−4.02,-0.41)
	Central Latin America	173017.76(159454.61,187101.06)	384033.63(357568.69,411853.66)	105.24(96.99,113.80)	151.79(141.33,162.79)	44.24(40.35,47.99)
	Central Sub-Saharan Africa	28247.45(25786.90,30696.44)	70727.44(65523.06,76565.40)	51.39(46.92,55.85)	51.65(47.85,55.92)	0.50(−2.35,3.25)
	East Asia	1147885.24(1002332.55,1288506.16)	1392547.97(1241282.77,1558662.32)	94.29(82.33,105.84)	94.55(84.28,105.83)	0.28(−4.13,4.63)
	Eastern Europe	232591.98(205338.23,264099.80)	244739.32(216483.38,276836.07)	102.69(90.66,116.60)	118.37(104.70,133.89)	15.26(12.45,17.94)
	Eastern Sub-Saharan Africa	107959.45(98238.09,118535.49)	220523.92(202100.83,239702.49)	56.58(51.48,62.12)	51.75(47.43,56.26)	−8.52(−10.53,-6.60)
	High-income Asia Pacific	386168.38(343656.54,431630.67)	747778.52(674045.57,832435.30)	222.73(198.21,248.95)	403.23(363.47,448.88)	81.04(74.94,87.22)
	High-income North America	305044.04(273290.30,342708.62)	512780.96(466659.46,564387.53)	108.40(97.11,121.78)	138.52(126.06,152.47)	27.79(22.41,33.23)
	North Africa and Middle East	226025.81(208422.99,245127.58)	422055.62(389156.96,456658.60)	66.64(61.45,72.27)	67.75(62.46,73.30)	1.66(−0.05,3.42)
	Oceania	7073.09(6576.33,7540.58)	15490.19(14650.92,16406.39)	107.99(100.40,115.12)	111.22(105.19,117.80)	2.99(0.65,5.47)
	South Asia	691952.16(607644.14,780839.73)	1355698.01(1203895.09,1512996.08)	63.28(55.57,71.41)	73.42(65.20,81.94)	16.01(12.88,19.16)
	Southeast Asia	423364.86(384441.26,464831.16)	804234.79(736667.92,876796.41)	90.95(82.59,99.85)	115.17(105.49,125.56)	26.63(23.56,29.60)
	Southern Latin America	73435.90(68870.24,78718.32)	98117.29(90839.92,107907.05)	148.24(139.02,158.90)	144.94(134.19,159.40)	−2.23(−6.56,6.41)
	Southern Sub-Saharan Africa	27258.59(24371.53,30080.58)	48804.40(44313.03,53405.94)	52.00(46.49,57.39)	60.77(55.18,66.50)	16.87(14.12,19.46)
	Tropical Latin America	164919.95(145257.59,185464.76)	278850.77(248463.67,313441.97)	108.11(95.22,121.58)	122.56(109.20,137.76)	13.37(9.01,17.62)
	Western Europe	503131.46(466769.32,541147.85)	608489.56(569044.47,647049.04)	130.88(121.42,140.77)	139.12(130.10,147.93)	6.29(4.50,8.23)
	Western Sub-Saharan Africa	111635.76(101453.35,122867.14)	241300.64(219739.46,264664.78)	57.79(52.52,63.61)	49.26(44.86,54.03)	−14.76(−16.40,-13.27)
DALYs	Global	12031276.33(9409789.62,14507855.38)	10641881.91(9398962.53,12121262.54)	225.57(176.42,272.01)	134.85(119.10,153.60)	−40.22(−49.46,-25.48)
	High SDI	1770611.40(1683805.04,1850531.98)	1591960.67(1476738.41,1698021.92)	201.31(191.44,210.40)	145.51(134.98,155.21)	−27.72(−31.46,-24.58)
	High-middle SDI	2675984.06(2099524.37,3179317.05)	1809134.10(1634520.36,2096840.68)	251.61(197.41,298.94)	138.73(125.34,160.80)	−44.86(−56.04,-27.46)
	Middle SDI	5166451.72(3272160.79,6399587.60)	3869941.55(3304695.26,4361938.82)	299.87(189.92,371.44)	158.05(134.97,178.14)	−47.29(−58.95,-22.90)
	Low-middle SDI	1883192.32(1378213.19,2530728.97)	2526898.96(2013968.54,3186820.97)	162.15(118.67,217.90)	131.53(104.83,165.88)	−18.88(−29.89,-5.06)
	Low SDI	524864.05(318474.50,841167.44)	833678.08(503946.81,1503254.11)	104.70(63.53,167.80)	74.61(45.10,134.53)	−28.74(−40.55,-14.02)
	Andean Latin America	86449.38(73688.55,103693.79)	126533.26(105431.04,151575.20)	227.54(193.95,272.93)	191.33(159.42,229.20)	−15.91(−34.88,3.42)
	Australasia	31758.66(30086.38,33458.15)	33262.24(30722.59,35750.44)	156.63(148.38,165.01)	107.43(99.23,115.47)	−31.41(−36.25,-26.55)
	Caribbean	75786.59(60139.75,86677.33)	96276.49(73558.55,118611.98)	214.74(170.40,245.60)	202.86(154.99,249.93)	−5.53(−21.34,14.38)
	Central Asia	74575.85(68063.95,82370.18)	125918.17(112903.97,139771.78)	107.59(98.20,118.84)	131.43(117.84,145.89)	22.15(5.07,39.19)
	Central Europe	268939.67(254525.00,283827.75)	189357.67(174635.90,203796.42)	214.99(203.47,226.90)	164.28(151.51,176.81)	−23.59(−29.79,-17.31)
	Central Latin America	181433.49(174449.43,189398.71)	392187.97(350308.76,440360.27)	110.36(106.11,115.20)	155.01(138.46,174.05)	40.47(25.45,57.63)
	Central Sub-Saharan Africa	38994.94(24216.78,71555.36)	74837.18(37073.15,182349.89)	70.95(44.06,130.19)	54.65(27.07,133.17)	−22.97(−48.07,19.37)
	East Asia	5374052.46(2858885.21,6883392.90)	2396954.74(1840650.49,2934106.74)	441.42(234.83,565.40)	162.75(124.98,199.22)	−63.13(−74.41,-38.73)
	Eastern Europe	443569.35(419105.50,465193.52)	494656.77(457049.39,532536.87)	195.84(185.04,205.39)	239.24(221.05,257.56)	22.16(12.99,32.75)
	Eastern Sub-Saharan Africa	150343.88(67641.52,342239.77)	234777.73(105025.54,589266.39)	78.79(35.45,179.35)	55.10(24.65,138.29)	−30.06(−41.97,-14.89)
	High-income Asia Pacific	577891.28(536256.21,635679.96)	485219.01(434133.76,531777.40)	333.31(309.30,366.64)	261.65(234.10,286.76)	−21.50(−28.79,-14.67)
	High-income North America	417065.39(401127.60,433045.30)	525515.54(495261.05,554719.65)	148.21(142.54,153.88)	141.96(133.79,149.85)	−4.21(−8.34,-0.80)
	North Africa and Middle East	499298.45(383573.24,680575.78)	433903.73(351654.09,547034.93)	147.20(113.08,200.65)	69.65(56.45,87.81)	−52.69(−66.87,-39.67)
	Oceania	17488.86(12752.86,22811.70)	31039.53(23596.39,40113.05)	267.01(194.70,348.27)	222.86(169.42,288.01)	−16.53(−34.89,8.39)
	South Asia	1640095.84(1113713.07,2409947.45)	2362778.66(1772137.15,3126992.84)	150.00(101.86,220.41)	127.95(95.97,169.34)	−14.70(−28.77,5.23)
	Southeast Asia	885900.64(731092.41,1148514.08)	1279431.08(1094265.56,1636990.90)	190.31(157.05,246.72)	183.22(156.70,234.42)	−3.73(−19.80,15.89)
	Southern Latin America	157011.50(145778.96,166179.21)	108157.31(101029.05,114847.37)	316.95(294.27,335.46)	159.77(149.24,169.66)	−49.59(−53.36,-45.49)
	Southern Sub-Saharan Africa	22233.31(19073.50,27737.93)	47011.48(39292.01,58929.68)	42.42(36.39,52.92)	58.54(48.93,73.38)	38.02(20.30,58.64)
	Tropical Latin America	341128.54(330820.37,351731.89)	460760.09(440239.46,479157.82)	223.62(216.86,230.57)	202.51(193.49,210.60)	−9.44(−13.71,-5.41)
	Western Europe	616322.44(589157.67,641599.54)	511251.42(473457.51,544420.85)	160.33(153.26,166.90)	116.89(108.25,124.47)	−27.10(−30.83,-24.02)
	Western Sub-Saharan Africa	130935.83(75880.92,259882.44)	232051.84(137193.50,464758.41)	67.79(39.28,134.54)	47.37(28.01,94.88)	−30.11(−45.50,-12.91)
Deaths	Global	374887.47(270973.34,465034.56)	352810.22(309015.35,401473.54)	7.03(5.08,8.72)	4.47(3.92,5.09)	−36.39(−47.81,-15.17)
	High SDI	55935.61(52505.74,58697.59)	59891.08(53245.55,63666.69)	6.36(5.97,6.67)	5.47(4.87,5.82)	−13.92(−20.68,-9.06)
	High-middle SDI	87851.98(66600.76,107274.68)	67000.72(58710.66,79143.00)	8.26(6.26,10.09)	5.14(4.50,6.07)	−37.80(−52.49,-11.86)
	Middle SDI	170253.56(94183.23,217750.11)	132719.98(109151.25,153400.99)	9.88(5.47,12.64)	5.42(4.46,6.26)	−45.15(−59.19,-11.13)
	Low-middle SDI	47620.80(32111.89,69056.37)	71891.63(55284.47,91825.64)	4.10(2.76,5.95)	3.74(2.88,4.78)	−8.73(−24.47,13.80)
	Low SDI	12953.89(6829.89,22375.99)	20995.81(11604.56,40674.81)	2.58(1.36,4.46)	1.88(1.04,3.64)	−27.28(−41.24,-13.13)
	Andean Latin America	1979.59(1671.60,2479.29)	3642.35(2950.93,4429.77)	5.21(4.40,6.53)	5.51(4.46,6.70)	5.70(−20.88,34.28)
	Australasia	1029.26(968.31,1090.60)	1320.84(1159.77,1434.84)	5.08(4.78,5.38)	4.27(3.75,4.63)	−15.96(−23.29,-9.01)
	Caribbean	1816.85(1488.23,2143.90)	2626.58(2037.27,3279.34)	5.15(4.22,6.07)	5.53(4.29,6.91)	7.51(−10.20,29.60)
	Central Asia	2148.48(1922.97,2409.14)	4005.69(3646.14,4454.07)	3.10(2.77,3.48)	4.18(3.81,4.65)	34.88(14.91,55.21)
	Central Europe	7787.51(7353.64,8239.74)	7012.27(6433.87,7557.84)	6.23(5.88,6.59)	6.08(5.58,6.56)	−2.28(−10.62,6.41)
	Central Latin America	4241.36(4105.09,4423.31)	11946.38(10601.28,13370.35)	2.58(2.50,2.69)	4.72(4.19,5.28)	83.03(62.55,106.01)
	Central Sub-Saharan Africa	893.58(466.92,1834.93)	1846.16(765.98,5027.35)	1.63(0.85,3.34)	1.35(0.56,3.67)	−17.07(−43.99,29.29)
	East Asia	191907.65(92729.85,251489.56)	95179.87(70278.39,119162.92)	15.76(7.62,20.66)	6.46(4.77,8.09)	−59.00(−72.76,-26.87)
	Eastern Europe	14361.64(13450.18,15194.09)	18199.46(16681.47,19688.43)	6.34(5.94,6.71)	8.80(8.07,9.52)	38.82(26.74,52.11)
	Eastern Sub-Saharan Africa	3675.90(1366.16,9333.07)	5580.10(2114.69,15463.46)	1.93(0.72,4.89)	1.31(0.50,3.63)	−32.02(−46.15,-16.77)
	High-income Asia Pacific	17904.22(16557.96,19797.38)	17741.78(14894.44,19535.03)	10.33(9.55,11.42)	9.57(8.03,10.53)	−7.35(−18.98,3.11)
	High-income North America	12540.15(11848.57,12964.87)	19705.08(17782.12,20833.24)	4.46(4.21,4.61)	5.32(4.80,5.63)	19.46(12.59,24.40)
	North Africa and Middle East	11473.87(7926.19,17666.80)	12261.22(9702.75,15966.22)	3.38(2.34,5.21)	1.97(1.56,2.56)	−41.82(−63.66,-21.69)
	Oceania	390.54(275.37,536.94)	700.89(502.96,961.80)	5.96(4.20,8.20)	5.03(3.61,6.91)	−15.60(−34.94,10.89)
	South Asia	42279.72(25791.86,67711.24)	67617.94(48401.37,93444.47)	3.87(2.36,6.19)	3.66(2.62,5.06)	−5.30(−24.39,25.90)
	Southeast Asia	23758.00(18821.78,33540.30)	37581.74(31535.11,51344.56)	5.10(4.04,7.21)	5.38(4.52,7.35)	5.45(−17.40,30.97)
	Southern Latin America	4547.67(4155.14,4844.03)	3521.74(3254.92,3745.76)	9.18(8.39,9.78)	5.20(4.81,5.53)	−43.33(−48.41,-37.64)
	Southern Sub-Saharan Africa	527.90(446.86,699.95)	1220.19(1007.87,1572.98)	1.01(0.85,1.34)	1.52(1.26,1.96)	50.88(26.73,76.67)
	Tropical Latin America	8105.80(7859.19,8381.65)	14034.53(13232.01,14630.88)	5.31(5.15,5.49)	6.17(5.82,6.43)	16.09(9.12,22.34)
	Western Europe	20414.36(19178.01,21267.72)	21913.14(19165.16,23395.64)	5.31(4.99,5.53)	5.01(4.38,5.35)	−5.66(−12.89,-0.43)
	Western Sub-Saharan Africa	3103.40(1545.04,6829.23)	5152.25(2595.40,11593.32)	1.61(0.80,3.54)	1.n(0.53,2.37)	−34.53(−51.09,-15.49)

Global age-standardized SAH incidence rates declined from 1990 to 2021, from 11.69 (95% UI 10.22–13.50) to 8.33 (95% UI 7.34–9.48) per 100,000. Sex differences showed consistently higher incidence rates in females than males (2021: females 8.17 vs. males 8.51). Among SDI regions, middle SDI regions exhibited the largest decline (from 14.85 to 9.16), with females in high SDI regions consistently showing higher incidence rates than males (2021: 8.72 vs. 6.94). Geographically, East Asia showed the most significant decline (from 17.74 to 7.89), while Andean Latin America maintained the highest incidence rate (2021: 12.29). Persistent sex disparities were observed in high-income Asia Pacific (2021: females 15.95 vs. males 11.93) and the Caribbean (females 11.93 vs. males 8.82).

The global prevalence of SAH increased significantly from 1990 to 2021, rising from 4.90 million (95% UI 4.42–5.39 million) to 7.85 million (95% UI 7.16–8.58 million). Stratified by SDI, high SDI regions increased from 1.23 million (95% UI 1.12–1.34 million) to 1.94 million (95% UI 1.78–2.09 million), with middle SDI regions showing the largest increase, from 1.54 million (95% UI 1.37–1.70 million) to 2.49 million (95% UI 2.26–2.75 million). Regionally, East Asia bore the heaviest burden, reaching 1.39 million (95% UI 1.24–1.56 million) in 2021, while South Asia exhibited the largest increase, from 0.69 million (95% UI 0.61–0.78 million) to 1.36 million (95% UI 1.20–1.51 million). Females consistently had higher prevalence than males (2021: females 4.31 million [95% UI 3.95–4.69 million] vs. males 3.54 million [95% UI 3.22–3.89 million]).

Age-standardized SAH prevalence rates showed significant regional and sex disparities. Globally, prevalence decreased from 109.90 (95% UI 99.05–121.56) to 92.17 (95% UI 84.08–100.60) per 100,000. Females had higher prevalence rates than males (2021: 97.88 vs. 85.52 per 100,000). High SDI regions had the highest prevalence (2021: 112.40 per 100,000), while East Asia showed the largest decline (from 108.08 to 70.06 per 100,000). Andean Latin America maintained the highest prevalence (2021: 172.03 per 100,000), while high-income Asia Pacific showed an increasing trend (from 191.54 to 193.60 per 100,000).

Global DALYs for SAH decreased from 12.03 million (95% UI 9.41–14.51 million) in 1990 to 10.64 million (95% UI 9.40–12.12 million) in 2021. High SDI regions decreased from 1.77 million (95% UI 1.68–1.85 million) to 1.59 million (95% UI 1.48–1.70 million), middle-high SDI regions from 2.68 million (95% UI 2.10–3.18 million) to 1.81 million (95% UI 1.63–2.10 million), and middle SDI regions from 5.17 million (95% UI 3.27–6.40 million) to 3.87 million (95% UI 3.30–4.36 million). Conversely, middle-low SDI regions increased from 1.88 million (95% UI 1.38–2.53 million) to 2.53 million (95% UI 2.01–3.19 million), and low SDI regions from 0.52 million (95% UI 0.32–0.84 million) to 0.83 million (95% UI 0.50–1.50 million). East Asia showed the most significant decline, from 5.37 million (95% UI 2.86–6.88 million) to 2.40 million (95% UI 1.84–2.93 million), while South Asia increased from 1.64 million (95% UI 1.11–2.41 million) to 2.36 million (95% UI 1.77–3.13 million). High-income North America also saw an increase, from 0.42 million (95% UI 0.40–0.43 million) to 0.53 million (95% UI 0.50–0.55 million).

Global age-standardized SAH DALY rates declined significantly from 275.85 (95% UI 213.22–335.43) to 125.20 (95% UI 110.54–142.61) per 100,000. Among SDI regions, high SDI regions had the lowest DALY rate in 2021 (91.84, 95% UI 86.03–97.38), while middle SDI regions showed the largest decline (from 446.97 to 143.43). East Asia exhibited the most significant improvement (from 601.42 to 116.37), while Oceania maintained the highest burden (285.62). Sex differences indicated lower DALY rates in females globally, though regions like Andean Latin America showed a reverse pattern.

Global SAH mortality decreased from 374,887 (95% UI 270,973–465,035) in 1990 to 352,810 (95% UI 309,015–401,474) in 2021. Sex differences were notable: in 2021, male deaths were 173,754 (95% UI 140,710–217,574), and female deaths were 179,056 (95% UI 156,258–208,098). High SDI regions reported 59,891 deaths (95% UI 53,246–63,667), while low SDI regions showed the largest increase (1990–2021: 12,954 to 20,996, a 62% rise). East Asia saw the most significant decline in deaths (from 191,908 to 95,180), while South Asia increased by 60% (from 42,280 to 67,618). Central Latin America and Tropical Latin America saw substantial increases of 182% (from 4,241 to 11,946) and 73% (from 8,106 to 14,035), respectively.

Global age-standardized SAH mortality rates declined significantly from 9.54 (95% UI 6.80–11.91) to 4.18 (95% UI 3.66–4.76) per 100,000. Middle SDI regions showed the largest decline (from 17.91 [95% UI 9.50–23.15] to 5.23 [95% UI 4.29–6.08]), while low SDI regions had the smallest decline (from 5.22 [95% UI 2.56–9.37] to 3.79 [95% UI 2.06–7.35]). East Asia exhibited the most significant improvement (from 26.47 [95% UI 12.55–34.94] to 4.71 [95% UI 3.49–5.87]). Males generally had higher mortality rates than females (2021: males 4.48 [95% UI 3.64–5.56] vs. females 3.91 [95% UI 3.41–4.55]), though high SDI regions showed a reverse pattern (females 2.97 [95% UI 2.63–3.18] vs. males 2.76 [95% UI 2.59–2.89]).

### 3.2 National SAH burden

**Figure 1:**
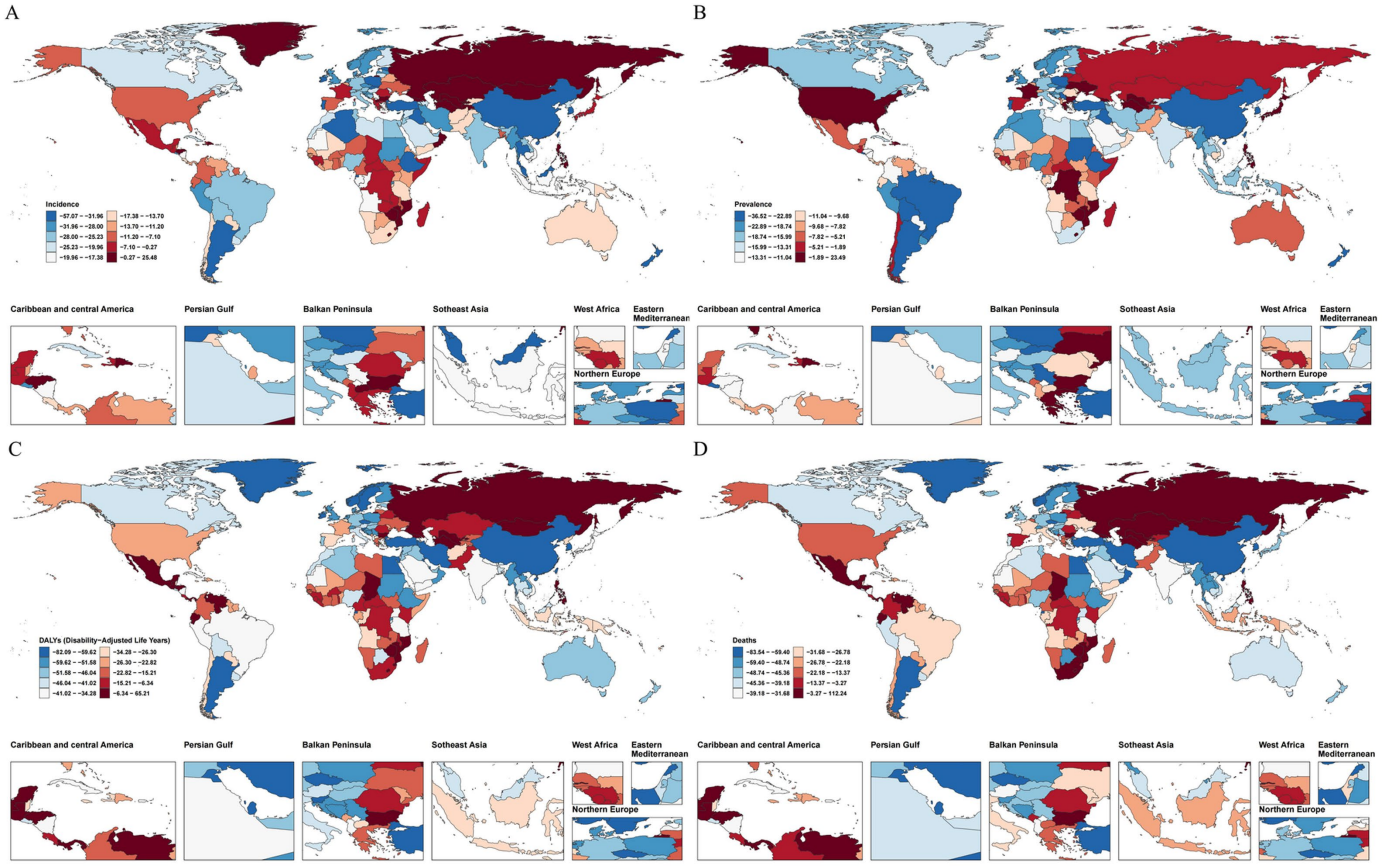
The global distribution of the age-standardized rates and AAPC for SHA age-standardized incidence **(A)**, prevalence **(B)**, DALYs **(C)**, and mortality **(D)** rates for SHA in 2021.

GBD data were used to visualize regional differences in age-standardized SAH incidence, prevalence, DALY, and mortality rates from 1990 to 2021 (Figure 1). Significant regional disparities in age-standardized SAH incidence rates were observed (Figure 1A). The top five regions with the largest declines included China (PC −56.50, 95% UI −60.17 to −53.32), Iraq (PC −40.93, 95% UI −44.63 to −36.99), Turkey (PC −39.16, 95% UI −44.08 to −33.46), Ireland (PC −39.10, 95% UI −43.70 to −33.80), and Poland (PC −37.51, 95% UI −40.17 to −34.52). The top five regions with the largest increases or smallest declines included Georgia (PC + 17.82%, 95% UI 10.03 to 26.70), Turkmenistan (PC + 25.23%, 95% UI 17.77 to 33.53), Zimbabwe (PC + 23.85%, 95% UI 15.63 to 33.69), the Philippines (PC + 25.04%, 95% UI 18.20 to 32.09), and the Solomon Islands (PC + 20.85%, 95% UI −0.91 to 41.09).

Age-standardized SAH prevalence rates showed significant regional disparities (Figure 1B). The top five regions with increases included the Philippines (PC 23.26%, 95% UI 19.08–26.86), Turkmenistan (PC 18.61%, 95% UI 13.22–23.82), Zimbabwe (PC 17.06%, 95% UI 12.36–22.17), Japan (PC 12.11%, 95% UI 8.82–15.62), and Greece (PC 6.50%, 95% UI 2.26–10.64). The top five regions with declines included Ethiopia (PC −31.40%, 95% UI −33.83 to −29.06), Ireland (PC −30.69%, 95% UI −33.51 to −27.34), Poland (PC −28.91%, 95% UI −30.94 to −26.87), Turkey (PC −28.84%, 95% UI −31.47 to −26.08), and Estonia (PC −28.74%, 95% UI −31.33 to −25.81).

Age-standardized SAH DALY rates exhibited significant regional disparities (Figure 1C). The top five regions with the largest declines included China (−81.28%, 95% UI −87.24 to −68.37), Turkey (−66.49%, 95% UI −79.10 to −52.18), Lebanon (−73.01%, 95% UI −82.43 to −60.16), Maldives (−69.44%, 95% UI −77.80 to −59.06), and Egypt (−64.81%, 95% UI −77.74 to −46.60). The top five regions with the largest increases or smallest declines included Zimbabwe (+64.56%, 95% UI 20.18 to 119.06), Uzbekistan (+56.77%, 95% UI 25.93 to 95.94), Turkmenistan (+41.70%, 95% UI 8.98 to 85.43), Lesotho (+39.66%, 95% UI −9.67 to 110.23), and Guatemala (+18.79%, 95% UI −0.90 to 40.71).

Age-standardized SAH mortality rates showed significant regional disparities (Figure 1D). The top five regions with increases included Uzbekistan (111.13%, 95% UI 60.68–170.21), Zimbabwe (51.55%, 95% UI 11.75–102.44), Turkmenistan (49.84%, 95% UI 13.21–99.34), Georgia (49.11%, 95% UI 11.66–105.55), and Mexico (30.58%, 95% UI 16.60–45.07). The top five regions with declines included China (−82.71%, 95% UI −88.71 to −67.93), Lebanon (−74.64%, 95% UI −84.90 to −60.57), South Korea (−77.90%, 95% UI −84.03 to −70.86), Singapore (−71.28%, 95% UI −74.37 to −68.52), and Kuwait (−72.42%, 95% UI −77.41 to −66.87).

### 3.3 Correlation of disease burden with socio-demographic index across 204 countries and territories

**Figure 2:**
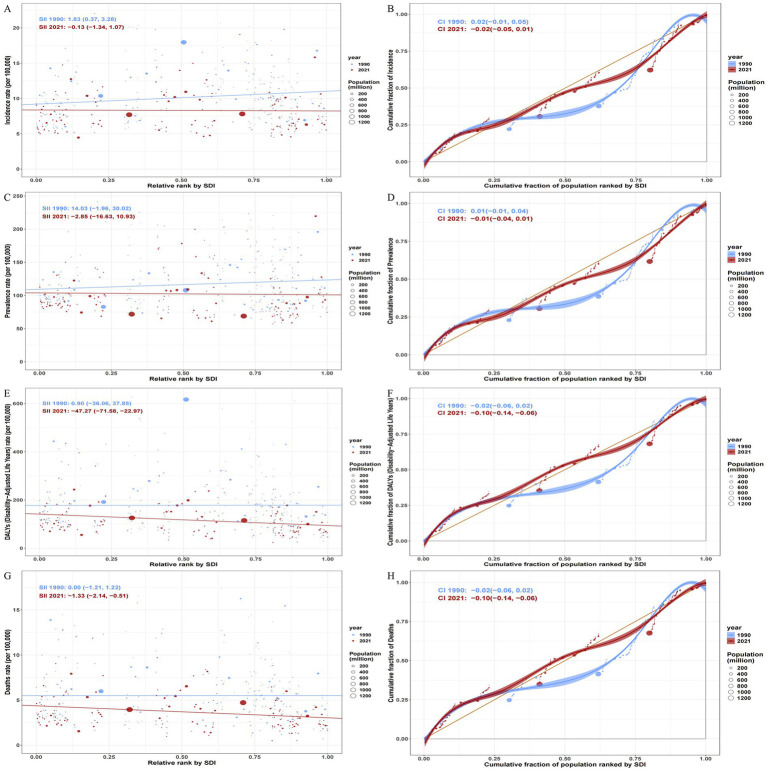
The correlation between the disease burden and regional SDl in 204 countries and regions. **(A)** Prevalence rate **(B)** Cumulative fraction of Incidence **(C)** Prevalence rate **(D)** Cumulative fraction of Prevalence **(E)** DALYs (Disability-Adjusted Life Years) rate **(F)** Cumulative fraction of DALYs(Disability-Adjusted Life Years) **(G)** Deaths rate **(H)** Cumulative fraction of Deaths.

Based on GBD data, we analyzed the correlation between age-standardized SAH incidence, prevalence, DALY, and mortality rates and SDI across 204 countries and territories (Figure 2). Spearman correlation analysis showed a weak negative correlation between SAH incidence and SDI (*r* = −0.0365, 95% CI −0.1659 to 0.0988), which was not statistically significant (*p* = 0.604). Scatter plots (Figure 2A) revealed significant heterogeneity in SAH incidence across SDI levels. High incidence rates (>15 per 100,000) were concentrated in Pacific Island nations (e.g., Kiribati, Solomon Islands) and some South American countries (e.g., Ecuador, Colombia), while low incidence rates (<5 per 100,000) were observed in high SDI countries (e.g., Italy, Bahrain) and some African countries (e.g., South Africa, Nigeria).

The estimated annual percentage change (EAPC) in incidence across 204 countries and territories in 2021 showed significant heterogeneity and a negative correlation with SDI (Figure 2B, right). High SDI countries (e.g., Japan, Germany, Sweden) generally exhibited more pronounced negative EAPC values (range: −0.21 to −3.70), while low SDI countries (e.g., Philippines, Zimbabwe) showed positive growth (range: 0.54 to 1.17). Specifically, the Philippines (EAPC = 1.17, SDI = 0.65) and Turkmenistan (EAPC = 1.04, SDI = 0.68) had the fastest incidence increases, while China (EAPC = −3.70, SDI = 0.72) and Iraq (EAPC = −2.17, SDI = 0.66) showed the largest declines. Spearman correlation analysis indicated that higher SDI levels were associated with more significant declines in disease burden (*ρ* = −0.62, *p* < 0.001). Additionally, countries with high baseline incidence rates (e.g., Solomon Islands, 24.22 per 100,000) showed flatter EAPC trends, potentially reflecting challenges in implementing prevention measures in low SDI regions (Figure 2B, left).

SAH prevalence showed a weak negative correlation with SDI (*r* = −0.0935, 95% CI −0.2168 to 0.0280), which was not statistically significant (*p* = 0.183). Scatter plots (Figure 2C) revealed significant regional disparities in prevalence, with Latin America (e.g., Ecuador: 199.8 per 100,000) and Western Pacific Island nations (e.g., Kiribati: 241.2 per 100,000) showing significantly higher prevalence than European countries (e.g., Germany: 93.3 per 100,000). Despite high prevalence in both high SDI countries (e.g., Japan: 219.6 per 100,000) and low SDI countries (e.g., Niger: 91.0 per 100,000), the overall correlation was weak, suggesting SDI is not a primary determinant of SAH prevalence. The EAPC in prevalence across 204 countries and territories (Figure 2D) showed significant positive growth in middle-low SDI countries like the Philippines (EAPC = 0.82), Turkmenistan (EAPC = 0.62), and Zimbabwe (EAPC = 0.57), indicating increasing disease burden. High SDI countries like Japan (EAPC = 0.41) and the United States (EAPC = 0.16) showed slower growth, while China (EAPC = −1.65) and Poland (EAPC = −1.27) exhibited significant negative EAPC values, suggesting effective prevention measures. High SDI countries (SDI ≥ 0.8, e.g., Switzerland: SDI = 0.93, EAPC = −0.37; Norway: SDI = 0.92, EAPC = −0.75) generally showed declining EAPC, likely linked to healthcare investments and public health policies. Low SDI countries (e.g., Central African Republic: SDI = 0.31, EAPC = −0.07) had high disease burdens but smaller EAPC declines, indicating limited healthcare system capacity. Outliers like Japan (SDI = 0.87, EAPC = 0.41) and Nauru (SDI = 0.63, EAPC = 0.04) suggest confounding factors such as aging populations or unique epidemiological characteristics.

SAH DALY rates showed a significant negative correlation with SDI (*r* = −0.3541, 95% CI −0.4594 to −0.2403, *p* = 2.472 × 10^−7^). This negative correlation (*r* = −0.35) indicates higher SAH burden in lower SDI countries, with lower burden in high SDI countries. Scatter plots (Figure 2E) showed that some low-income countries (e.g., Haiti, Bolivia, Marshall Islands) had significantly higher DALY rates than the global average (e.g., Haiti: 434.98 per 100,000), while high SDI countries (e.g., Switzerland, Sweden, Israel) had lower rates (e.g., Switzerland: 46.55 per 100,000). The EAPC in DALYs across 204 countries and territories in 2021 (Figure 2F) showed a significant negative correlation with DALYs (*r* = −0.42, *p* < 0.001), indicating slower EAPC growth or declines in regions with higher disease burden. For instance, Zimbabwe (EAPC = 2.38, DALYs = 184.88) and Georgia (EAPC = 1.82, DALYs = 244.00) had high EAPC values, while China (EAPC = −6.29, DALYs = 115.49) and South Korea (EAPC = −4.41, DALYs = 107.27) showed significant declines. EAPC was also negatively correlated with SDI (*r* = −0.38, *p* < 0.001), with high SDI countries like Switzerland (SDI = 0.93, EAPC = −2.61) and Norway (SDI = 0.92, EAPC = −3.21) showing lower EAPC values than middle-low SDI countries, suggesting that higher socioeconomic development facilitates effective disease burden reduction through robust healthcare systems and interventions.

SAH mortality rates showed a significant negative correlation with SDI (*r* = −0.3058, 95% CI −0.4098 to −0.1833, *p* = 9.627 × 10^−6^). This weak-to-moderate negative correlation (*r* = −0.31) indicates higher SAH mortality in lower SDI countries. Scatter plots (Figure 2G) showed that low-income countries like the Marshall Islands (12.31 per 100,000), Haiti (12.78 per 100,000), and Mongolia (12.41 per 100,000) had significantly higher mortality rates than high SDI countries (e.g., Switzerland: 1.66 per 100,000; Kuwait: 0.51 per 100,000). Oceania Island nations (e.g., Solomon Islands: 11.68 per 100,000) and South Asia (e.g., Bangladesh: 7.93 per 100,000) had heavy SAH burdens, while high-income European countries (e.g., Sweden: 1.81 per 100,000) had lower mortality, suggesting disparities in healthcare access and risk factor management (e.g., hypertension). The EAPC in mortality across 204 countries and territories in 2021 (Figure 2H) showed that high SDI countries (SDI > 0.8, e.g., Switzerland, Norway) exhibited more pronounced negative EAPC values (*β* = −2.55, 95% CI −3.12 to −1.98), indicating faster mortality declines, while low SDI countries (SDI < 0.5, e.g., Chad, Niger) often showed positive EAPC values (e.g., Georgia: EAPC = 2.12), suggesting rising mortality. High-income European countries (e.g., Sweden: EAPC = −2.15) showed significantly larger EAPC declines than sub-Saharan Africa (e.g., Zimbabwe: EAPC = 1.55). East Asia displayed polarization, with China showing the largest decline (EAPC = −5.67) and Mongolia a slight increase (EAPC = 0.18).

### 3.4 Inequality analysis

**Figure 3:**
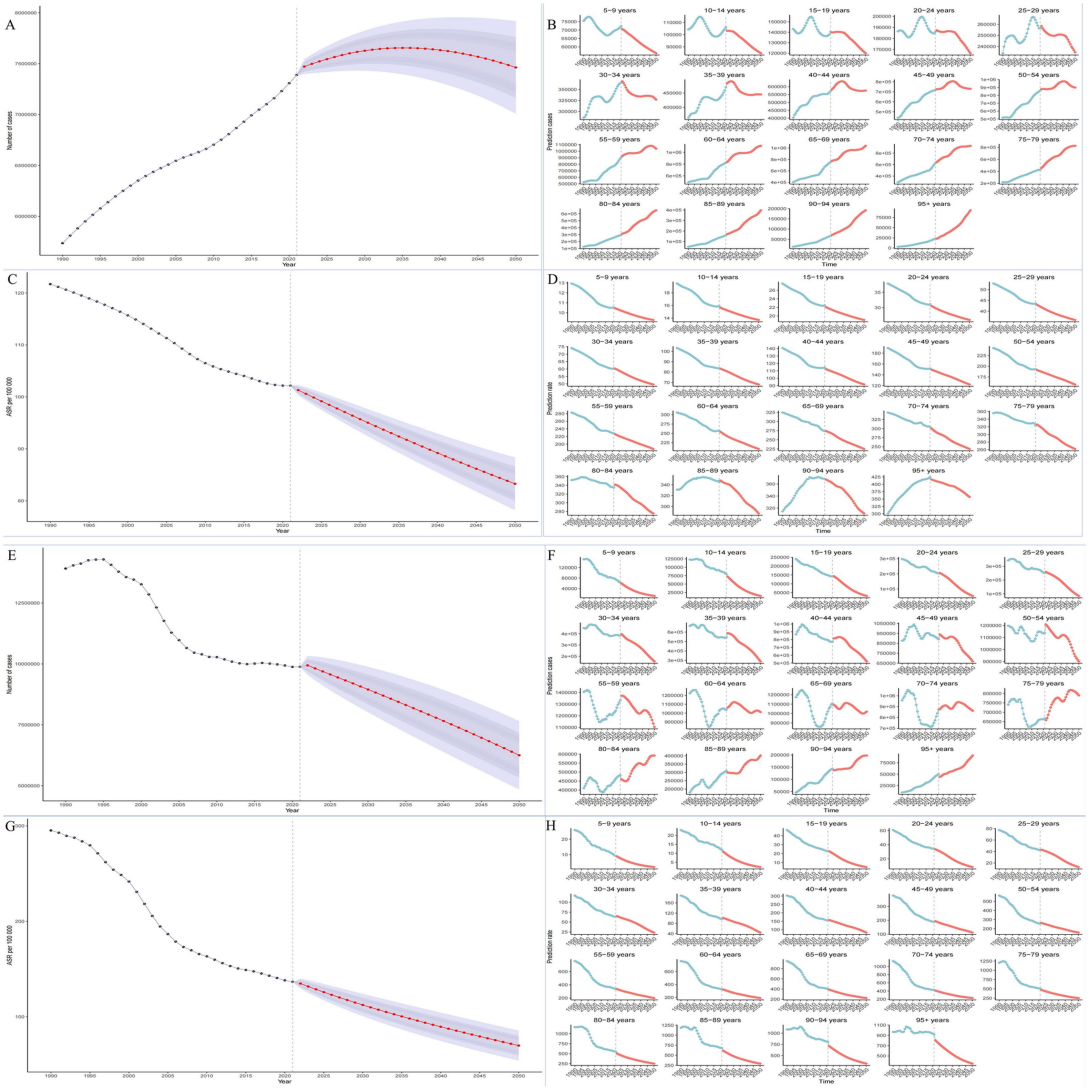
Analysis of health inequality. **(A)** Number of cases **(B)** Prediction cases **(C)** ASR Per **(D)** Prediction rate **(E)** Number of cases **(F)** Prediction cases **(G)** ASR Per **(H)** Prediction cases.

Socioeconomic inequality analysis of SAH incidence (regression curves; Figure 3A) showed that in 1990, the Slope Index of Inequality (SII) was 1.83 (95% CI 0.37–3.28), indicating a significant socioeconomic gradient with higher incidence among higher socioeconomic groups. By 2021, the SII turned negative (−0.13, 95% CI −1.34 to 1.07), suggesting a reversal where lower-income groups may face higher SAH incidence, though the confidence interval included zero, indicating no statistical significance.

Socioeconomic inequality analysis of SAH incidence (1990–2021, concentration curves, Figure 3B) showed that in 1990, the Concentration Index (CII) was 0.02 (95% CI −0.01 to 0.05), suggesting a slight but non-significant pro-rich distribution (CII > 0). By 2021, the CII turned negative (−0.02, 95% CI −0.05 to 0.01), indicating a shift in SAH burden toward lower-income groups, though this change was not statistically significant.

Using GBD data and regression curve analysis (Figure 3C), the SII for SAH prevalence in 1990 was 14.03 (95% CI −1.96 to 30.02), suggesting higher prevalence among higher socioeconomic groups, but the confidence interval included zero, indicating no statistical significance. By 2021, the SII decreased to −2.85 (95% CI −16.63 to 10.93), suggesting slightly higher prevalence among lower socioeconomic groups, though still not statistically significant.

Socioeconomic inequality analysis of SAH prevalence (1990–2021, concentration curves, Figure 3D) showed that in 1990, the CII was 0.014 (95% CI −0.008 to 0.036), near zero with a confidence interval crossing zero, indicating a relatively equitable distribution across socioeconomic groups. By 2021, the CII turned to −0.013 (95% CI −0.036 to 0.012), suggesting a slight shift toward lower-income groups, but with limited statistical significance due to the confidence interval crossing zero.

Socioeconomic inequality in SAH disease burden (DALYs) from 1990 to 2021 showed significant changes (regression curves; Figure 3E). In 1990, the SII was 0.90 (95% CI −36.06 to 37.85), indicating no significant association with socioeconomic status (confidence interval included zero). By 2021, the SII decreased to −47.27 (95% CI −71.58 to −22.97), indicating a significant shift in disease burden toward lower-income groups (SII negative, confidence interval excluding zero).

Socioeconomic inequality in SAH DALYs (concentration curves, Figure 3F) showed that in 1990, the CII was −0.02 (95% CI −0.06 to 0.02), suggesting a relatively equitable distribution (CII near zero, confidence interval crossing zero). By 2021, the CII decreased to −0.10 (95% CI −0.14 to −0.06), indicating a significant concentration of disease burden among lower-income groups (confidence interval excluding zero).

Socioeconomic inequality trends in SAH mortality (1990–2021, regression curves, Figure 3G) showed that in 1990, the SII was 0.00 (95% CI −1.21 to 1.22), indicating no significant association with socioeconomic status (regression slope near zero, confidence interval crossing zero). By 2021, the SII decreased to −1.33 (95% CI −2.14 to −0.51), suggesting significantly higher SAH mortality among socioeconomically disadvantaged groups (difference of 1.33 deaths per 100,000 person-years).

Socioeconomic inequality in SAH mortality (concentration curves, Figure 3H) showed that in 1990, the CII was −0.02 (95% CI −0.06 to 0.02), near zero with a confidence interval crossing zero, indicating a relatively equitable distribution across socioeconomic groups. By 2021, the CII decreased significantly to −0.10 (95% CI −0.14 to −0.06), with the confidence interval entirely negative, indicating a pronounced concentration of SAH mortality among lower socioeconomic groups, with increased inequality.

### 3.5 SAH prevencence and DALYs trends and projections (2050)

**Figure 4:**
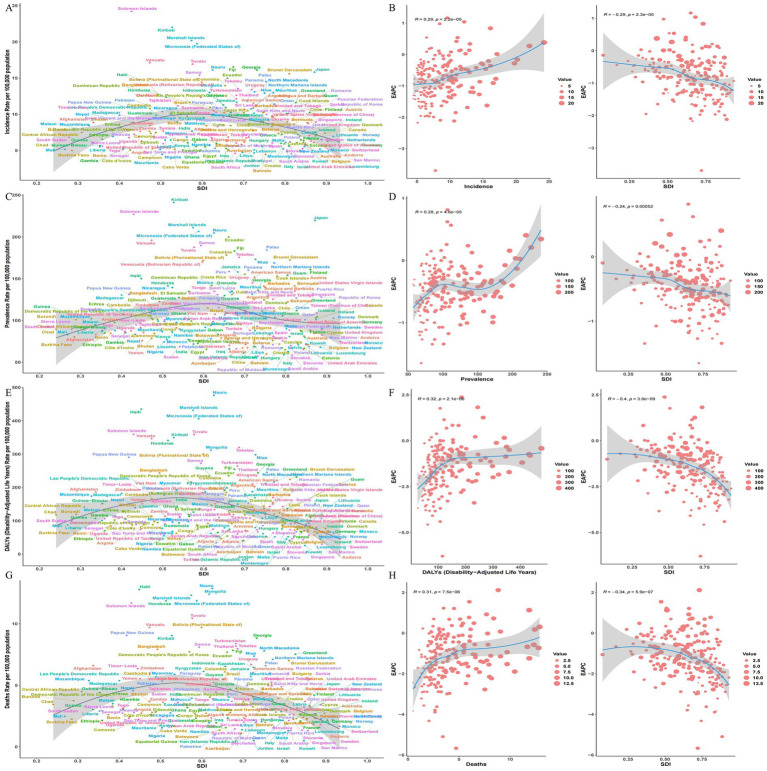
SAH prevencence and DALYs trends and projections (2050). **(A)** Incidence Rate per 100,000 population **(B, D, F, H)**. EPAC **(C)** Prevalence Rate per 100,000 population **(E)** DALYs (Disability-Adjusted Life Years) Rate per 100,000 population **(G)** Deaths Rate per 100,000 population.

Projected trends indicate an initial increase followed by a gradual decline in the number of prevalent SAH cases from 2022 to 2050 (Figure 4A); age-stratified analysis (Figure 4B) shows declining prevalence counts among individuals aged <34 years, an initial rise followed by a decline in the 34–59 age group, and an increasing trend among those aged ≥60 years. The overall prevalence rate demonstrates a declining trajectory (Figure 4C), consistently observed across all age groups (Figure 4D). Both DALY counts and DALY rates exhibit overall declining trends (Figures 4E,G); age-specific projections for DALY counts (Figure 4F) reveal a decrease in the <39 age group (2022–2050), an S-shaped decline in the 40–59 cohort, minimal net change between 2022 and 2050 in the 60–74 group indicating relative stability, and an increase among those aged ≥75 years. The DALY rate declined across all age groups (Figure 4H). Collectively, these findings demonstrate that the future burden of SAH will disproportionately shift toward the older adults population, necessitating targeted policy adaptations.

## 4 Discussion

### 4.1 National SAH burden analysis

Regions with significant declines, such as China and Iraq, likely benefited from improved hypertension management, smoking cessation policies, and enhanced healthcare access (e.g., China’s “Healthy China 2030” initiative) (8). Regions with increases (e.g., Georgia, Turkmenistan) require attention to uncontrolled hypertension, smoking rates, and limited healthcare resources (9). Middle-high SDI countries (e.g., Ireland, Poland) showed notable declines, suggesting a positive correlation between economic development and SAH prevention, though specific policy analyses are needed. Wide uncertainty intervals in some regions (e.g., Solomon Islands) reflect data uncertainty, necessitating strengthened local epidemiological surveillance. High-growth regions (e.g., Philippines, Turkmenistan) may be linked to uncontrolled hypertension, rising smoking rates, or lack of early interventions due to limited healthcare resources, requiring enhanced primary prevention (10). Significant declines in regions like Ethiopia and Ireland likely reflect improvements in stroke management systems (e.g., aneurysm screening, smoking cessation policies) or rising SDI (11). China’s substantial decline (PC −36.16%) highlights the effectiveness of public health measures (e.g., salt intake control, widespread hypertension treatment), serving as a model for other countries. Significant DALY rate reductions in China and Turkey may be attributed to advancements in neurosurgical techniques (e.g., widespread endovascular treatment), optimized hypertension management, and improved early diagnosis. High SDI countries (e.g., Maldives) demonstrate effective healthcare investments and public health policies. Rising DALY rates in Zimbabwe and Uzbekistan suggest healthcare system vulnerabilities (e.g., limited stroke unit coverage) or ineffective risk factor interventions (e.g., low hypertension control rates). SDI-stratified analysis showed a median DALY decline of −46.92% in high SDI countries compared to −27.48% in low SDI countries, reflecting the impact of unequal healthcare resource distribution. High growth in Central Asia (e.g., Uzbekistan, Turkmenistan) and Africa (e.g., Zimbabwe) may be linked to inadequate hypertension control, limited healthcare resources, and improved diagnostic capacity, necessitating targeted stroke prevention. Significant improvements in East Asia (e.g., China, South Korea, Singapore) and Gulf countries (e.g., Kuwait) likely stem from medical advancements (e.g., endovascular treatment) and risk factor management (e.g., hypertension screening) (12). Global trends indicate declines in high SDI countries (e.g., Europe), with significant heterogeneity in low SDI countries, underscoring the need for balanced healthcare resource allocation.

### 4.2 Correlation of disease burden with socio-demographic index across 204 countries and territories

The weak correlation between SAH incidence and SDI supports the multifactorial etiology of SAH, necessitating targeted hypertension control and healthcare resource allocation in high-incidence regions rather than relying solely on SDI-related socioeconomic improvements. The EAPC in incidence and its correlation with SDI highlight the need for differentiated public health strategies, with low SDI countries requiring enhanced healthcare resource allocation and primary prevention, and high SDI countries addressing emerging risks like aging populations. SDI’s mediating role suggests that coordinated development in education, economy, and healthcare infrastructure may reduce disease burden through multiple pathways (e.g., widespread screening, treatment access). From a global health equity perspective, these findings underscore health inequalities across SDI gradients, calling for international support for low- and middle-income countries.

The weak correlation between SAH prevalence and SDI suggests that non-socioeconomic factors (e.g., hypertension management, healthcare access, genetic predisposition) primarily drive disease burden rather than SDI. Future interventions should optimize stroke prevention systems in high-risk regions (e.g., Pacific Island nations). High prevalence in countries like Japan and Palau may be linked to genetic predispositions (e.g., hyperfibrinogenemia) or diagnostic capacity differences, warranting further research. The EAPC in prevalence and its correlation with SDI highlight the need for low SDI countries to prioritize disease surveillance and primary healthcare, while high SDI countries focus on chronic disease management. SDI-EAPC gradients underscore global healthcare resource disparities, necessitating international cooperation to bridge gaps. Future research should integrate specific disease profiles and interventions to elucidate EAPC heterogeneity.

The correlation between DALY rates and SDI suggests the need for enhanced SAH prevention (e.g., hypertension management) and emergency care capacity in low SDI regions. High SDI countries likely benefit from robust stroke care systems (e.g., early diagnosis, neurosurgical interventions), while inadequate hypertension control or poor healthcare access in low SDI regions may elevate SAH incidence. Global health aid should prioritize cerebrovascular disease prevention in high-risk countries (13).

The EAPC in incidence and its correlation with SDI indicate that middle-low SDI countries require strengthened healthcare resource allocation to address rising disease burdens, while high SDI countries’ experiences can inform disease burden management (e.g., early screening, preventive interventions). Further exploration of SDI-EAPC drivers, such as healthcare access and education levels, is needed.

The negative correlation between SAH mortality and SDI likely reflects inadequate cerebrovascular disease prevention, limited emergency neurosurgical services, or diagnostic capacity in low SDI regions. Prioritizing primary prevention (e.g., hypertension control) and healthcare system development in these regions is critical.

### 4.3 Inequality analysis

The shift in SII for SAH incidence from positive to negative from 1990 to 2021 may reflect reduced SAH burden among high-income groups in high-income countries through interventions like aneurysm screening and hypertension management, while low-income regions face rising burdens due to limited healthcare resources (14). Strengthening cerebrovascular disease prevention systems, particularly hypertension control and emergency neurosurgical services, in low-income regions is essential. The wide confidence interval for 2021 SII suggests the need for larger sample sizes to validate findings, with further stratification by region-specific data (e.g., SDI).

The shift in CII for SAH incidence from positive to negative suggests increasing influence of socioeconomic factors on SAH risk distribution, with rising relative risk among lower-income groups. Enhancing primary cerebrovascular disease prevention and emergency healthcare coverage for low-income populations is needed to address health inequalities.

The shift in SII for SAH prevalence from positive to negative suggests a possible reversal in socioeconomic inequality patterns, though further research is needed to confirm this trend. Despite non-significant changes, attention to SAH risk among socioeconomically disadvantaged groups and optimized healthcare resource allocation are warranted. Comprehensive analysis incorporating other health inequality metrics (e.g., Relative Index of Inequality, RII) and potential influencers (e.g., healthcare access, hypertension control) is needed.

The shift in SAH prevalence inequality suggests that from no significant socioeconomic gradient in 1990, a slight tendency toward lower-income groups emerged by 2021, possibly reflecting unequal healthcare resource distribution or accumulation of risk factors (e.g., inadequate hypertension management) among disadvantaged groups. Strengthening primary SAH prevention (e.g., blood pressure control) and early diagnostic access in low-income regions is critical to reducing health inequalities (14).

The increased negative SII for SAH DALYs in 2021 indicates a significant shift in disease burden toward socioeconomically disadvantaged groups, likely linked to inadequate hypertension management and poor healthcare access. Enhancing stroke prevention (e.g., blood pressure screening) and emergency healthcare coverage for low-income populations is essential to reduce health inequalities. The narrower confidence interval for 2021 SII compared to 1990 reflects improved GBD data quality, supporting conclusion reliability.

The negative shift in CII for SAH DALYs indicates increasing disease burden concentration among lower socioeconomic groups, likely due to disparities in healthcare access, risk factor management (e.g., inadequate hypertension control), or uneven prevention coverage. Prioritizing primary healthcare and stroke prevention programs for low-income groups is critical to reducing health inequities, consistent with socioeconomic gradient changes in other non-communicable diseases in GBD studies, emphasizing health equity as a core public health goal.

The negative SII for SAH mortality in 2021, with a significant confidence interval, indicates a concentration of SAH burden among low-income or low-education groups, likely due to inadequate hypertension management and poor healthcare access. Strengthening primary SAH prevention (e.g., blood pressure control) and emergency care systems for disadvantaged groups is essential to reduce health inequalities. The temporal SII trend aligns with other GBD studies, supporting heterogeneity in socioeconomic gradients’ impact on stroke subtypes.

The negative shift in CII for SAH mortality (increased absolute value) indicates increasing disease burden concentration among lower-income groups, likely linked to socioeconomic disparities in healthcare access and risk factor management (e.g., inadequate hypertension control). These findings call for enhanced early screening, blood pressure control, and emergency healthcare access for low-income groups to reduce health inequalities. The CII trend from 1990 to 2021 reflects a regression in global health equity for cerebrovascular diseases, necessitating integration into Sustainable Development Goals (SDG) monitoring frameworks.

## 5 Conclusion

This study reveals a complex global landscape for SAH from 1990 to 2021, with absolute case numbers increasing significantly (incidence from 508,789 [95% UI 441,504–587,616] to 697,486 [95% UI 614,334–795,785]; prevalence from 4.90 million [95% UI 4.42–5.39 million] to 7.85 million [95% UI 7.16–8.58 million]). However, age-standardized incidence, prevalence, DALY, and mortality rates declined significantly (incidence from 11.69 to 8.33 per 100,000; DALYs from 275.85 to 125.20 per 100,000; mortality from 9.54 to 4.18 per 100,000). East Asia showed the most significant declines (e.g., incidence from 17.74 to 7.89 per 100,000), while Andean Latin America maintained the highest burden (2021 prevalence: 172.03 per 100,000). Socioeconomic inequalities worsened, with 2021 disease burden significantly concentrated among lower-income groups (DALY SII: −47.27 [95% CI −71.58, −22.97]; mortality SII: −1.33 [95% CI −2.14, −0.51]). Persistent sex differences showed higher female incidence and prevalence but lower DALY and mortality rates. Middle SDI regions had the largest case number increases, yet the most significant age-standardized rate declines. National-level analysis revealed significant heterogeneity, with China showing the largest declines in incidence (−56.50%) and mortality (−82.71%), while Turkmenistan and Zimbabwe exhibited rising burdens. SAH burden showed weak negative correlations with SDI, with stronger correlations for DALYs (*r* = −0.3541, *p* < 0.001) and mortality (*r* = −0.3058, *p* < 0.001). The increase in absolute cases aligns with global population growth and aging, as older populations are more susceptible to SAH due to aneurysms. Declining age-standardized rates align with previous GBD findings, reflecting advancements in risk factor management (e.g., hypertension control) and diagnostic/treatment technologies. East Asia’s significant declines likely stem from robust healthcare systems and widespread hypertension screening, as seen in China and Japan. Conversely, high burdens in Andean Latin America and Oceania align with reports of limited healthcare access and uncontrolled risk factors (e.g., smoking, alcohol use). Higher female incidence and prevalence align with studies on hormonal factors and aneurysm formation, while lower female mortality may reflect better post-SAH care or differences in case severity. The shift in disease burden toward low SDI regions and lower-income groups is a novel finding, contrasting earlier studies suggesting higher SAH incidence in high-income groups due to better detection. This reversal highlights increasing inequalities driven by disparities in healthcare access and risk factor management. These findings underscore the need for targeted interventions in high-burden regions like Andean Latin America and Oceania, where incidence and prevalence remain high. Declining age-standardized rates suggest that prevention measures like hypertension management and smoking cessation are effective and should be expanded in low-middle SDI regions. The increasing burden among lower-income groups highlights the urgency of addressing socioeconomic barriers to healthcare access, including diagnostic imaging and neurosurgical care. Sex-specific strategies in high SDI regions (e.g., high-income Asia Pacific) could focus on aneurysm screening due to higher female incidence. However, the weak correlation between SDI and incidence/prevalence suggests local risk factors (e.g., genetic predisposition, environmental exposures) require further study. Future clinical trials should validate the effectiveness of region-specific interventions, particularly in low SDI regions with rising DALYs indicating unmet needs. Future research should explore drivers of increasing SAH burden in low SDI regions, including untreated hypertension, smoking, and limited emergency care. Longitudinal cohort studies in high-burden regions like Andean Latin America could elucidate region-specific risk factors. The socioeconomic shift in disease burden warrants investigation into social determinants of health, such as income inequality and healthcare infrastructure, and their impact on SAH outcomes. Geographic heterogeneity in Pacific Island nations due to genetic and environmental factors merits further study. The impact of aging populations on SAH trends, particularly in high SDI countries like Japan with rising prevalence, should be assessed. Advanced modeling techniques, such as machine learning, could enhance SAH burden prediction and guide resource allocation. This study leverages GBD data spanning 204 countries and territories over three decades, providing reliable estimates of SAH burden and trends. The use of age-standardized metrics and socioeconomic inequality indices (SII, CII) enhances comparability and highlights disparities. Regional and sex-specific analyses offer nuanced insights into global differences. However, limitations include potential biases from GBD data quality variations, particularly in low SDI regions, where underreporting or misdiagnosis may occur. The study did not account for specific risk factors like aneurysm prevalence or lifestyle, limiting causal inference. Weak statistical significance in some inequality analyses (e.g., incidence and prevalence CII) suggests caution in interpreting socioeconomic trends. Observational study design precludes definitive conclusions on intervention effectiveness. In conclusion, this study elucidates the complex global SAH landscape from 1990 to 2021, with increasing absolute case numbers but declining age-standardized rates. Significant regional and socioeconomic disparities, with rising burdens in low SDI regions and lower-income groups, emphasize the need for targeted public health strategies, improved healthcare access, and further research into region-specific risk factors to mitigate the global SAH burden and address emerging inequalities.

## Data Availability

The original contributions presented in the study are included in the article/supplementary material, further inquiries can be directed to the corresponding authors.

## Footnotes

1
https://vizhub.healthdata.org/gbd-results/

2
https://folk.ntnu.no/andrerie/software.html
